# Characterizing the Process Physics of Ultrasound-Assisted Bioprinting

**DOI:** 10.1038/s41598-019-50449-w

**Published:** 2019-09-25

**Authors:** Parth Chansoria, Rohan Shirwaiker

**Affiliations:** 1 Edward P. Fitts Department of Industrial and Systems Engineering, North Carolina State University, Raleigh, NC 27695 United States of America; 2 Comparative Medicine Institute, North Carolina State University, Raleigh, NC 27695 United States of America; 3 Joint Department of Biomedical Engineering, North Carolina State University and University of North Carolina at Chapel Hill, Raleigh, NC 27695 United States of America

**Keywords:** Biomedical engineering, Mechanical engineering

## Abstract

3D bioprinting has been evolving as an important strategy for the fabrication of engineered tissues for clinical, diagnostic, and research applications. A major advantage of bioprinting is the ability to recapitulate the patient-specific tissue macro-architecture using cellular bioinks. The effectiveness of bioprinting can be significantly enhanced by incorporating the ability to preferentially organize cellular constituents within 3D constructs to mimic the intrinsic micro-architectural characteristics of native tissues. Accordingly, this work focuses on a new non-contact and label-free approach called ultrasound-assisted bioprinting (UAB) that utilizes acoustophoresis principle to align cells within bioprinted constructs. We describe the underlying process physics and develop and validate computational models to determine the effects of ultrasound process parameters (excitation mode, excitation time, frequency, voltage amplitude) on the relevant temperature, pressure distribution, and alignment time characteristics. Using knowledge from the computational models, we experimentally investigate the effect of selected process parameters (frequency, voltage amplitude) on the critical quality attributes (cellular strand width, inter-strand spacing, and viability) of MG63 cells in alginate as a model bioink system. Finally, we demonstrate the UAB of bilayered constructs with parallel (0°–0°) and orthogonal (0°–90°) cellular alignment across layers. Results of this work highlight the key interplay between the UAB process design and characteristics of aligned cellular constructs, and represent an important next step in our ability to create biomimetic engineered tissues.

## Introduction

3D bioprinting technology is playing a key role in the field of tissue engineering and regenerative medicine to fabricate patient-specific engineered tissues^1–5^. The range of bioprinting modalities based on extrusion-based^6^, inkjet-based^7,8^, laser-based^9,10^ and vat photopolymerization-based^11,12^ principles offers the flexibility to process a wide range of bioinks containing autologous, allogenic or xenogeneic cells for creating different types of complex tissues for clinical, diagnostic and research applications^13^. Through the automation of material handling, tool path control, and crosslinking mechanisms, the variability in bioprinting processes can be significantly reduced, thereby enabling reproducibility^5^.

While bioprinting is highly effective in recapitulating the macro-geometry of the intended tissues, there is a scope to improve the capabilities to enable biomimicry of tissues’ micro-architectural characteristics. This is especially critical for tissues such as ligaments, tendons, and cardiac muscle that possess an intrinsic anisotropy of the cellular and extracellular matrix (ECM) components, which imparts highly specialized biomechanical characteristics specific to their location and function^14^. For example, the ECM in tendons has a predominantly linear orientation for resistance to tensile stresses, while that in annulus fibrosus has a crisscross alignment for resistance to compressive and hoop stresses^14^. Current bioprinting approaches typically utilize homogeneous cell distributions within the constitutive bioinks, which can result in unguided ECM formation from the cells during construct maturation. Herein, one effective strategy is to induce tissue-specific cellular alignment within the bioinks during bioprinting to promote aligned ECM production through the inter-cellular signaling that ensues, thereby mimicking the relevant biomechanical properties^14,15^. In addition to providing clinical alternatives, such biomimetically engineered tissues would also find applications in drug diagnostics^16,17^ and tissue growth and disease modeling studies^18–21^, among others.

**Figure 1:**
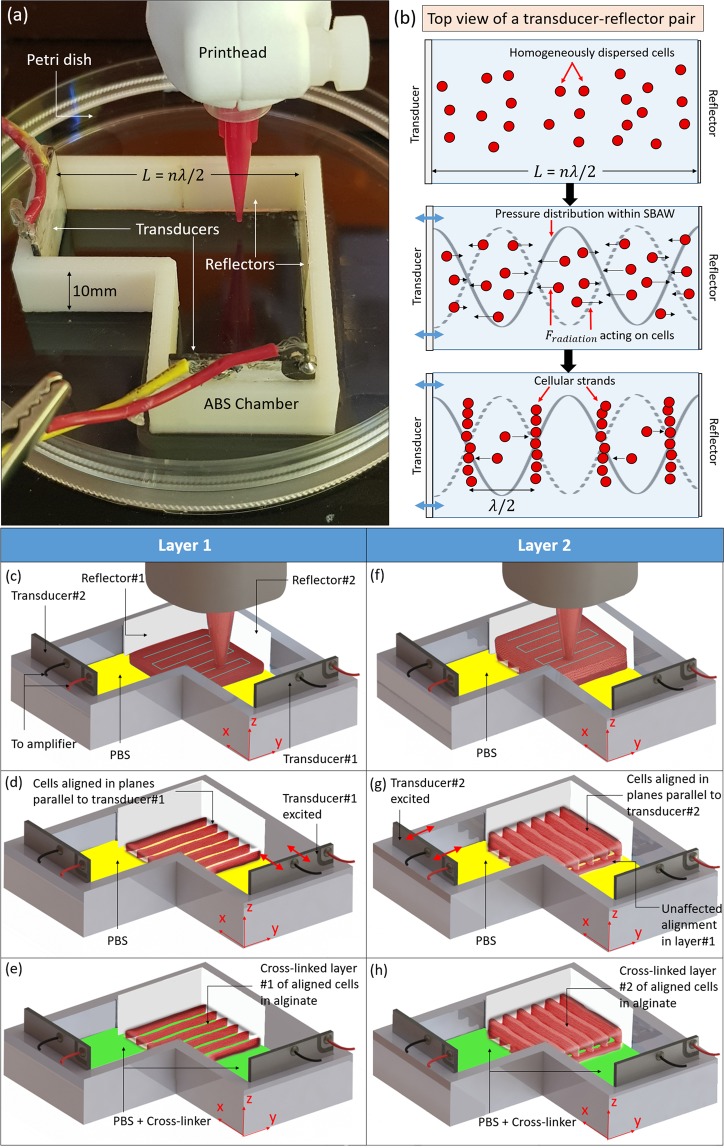
Ultrasound-assisted bioprinting. **(a)** The cross-patterning ultrasound alignment chamber that contains orthogonally arranged transducer-reflector pairs. **(b)** Schematic representation of the SBAW generated due to transducer excitation, and the resulting acoustic pressure distribution which exerts *F*_*radiation*_ on the cells to align them at the pressure nodes of the SBAW. **(c)** The bioink is printed as adjacent strands (blue lines depict that rectilinear pattern of deposition) into the chamber pre-filled with PBS buffer to constitute the first layer of the construct. The MG63 are homogeneously distributed across this layer. **(d)** Transducer #1, when excited using a sinusoidal voltage signal, vibrates along its thickness (along x-axis) to align the cells along nodal planes parallel to the transducer surface (y-z plane). **(e)** The alginate is gradually crosslinked by introducing the crosslinker (CaCl_2_) to entrap the aligned cells within the first layer. **(f)** The second layer of bioink is printed on top of the crosslinked first layer after aspirating all the fluid (PBS + CaCl_2_) in the chamber and adding fresh PBS. **(g)** Transducer#2, when excited using a sinusoidal voltage signal, vibrates along its thickness (along y-axis) to impart an orthogonal cellular alignment (x-z plane) relative to the first layer (0°–90° alignment). **(h)** The crosslinker (CaCl_2_) is introduced to gradually crosslink the second alginate layer while entrapping the aligned cells. Note that since the viscosity of crosslinked alginate is several orders of magnitude higher than its uncrosslinked solution counterpart^71^, the alignment of cells entrapped within the crosslinked first layer is not affected by transducer excitation during printing and alignment of the subsequent layer. To achieve parallel alignment (0°–0°) across layers, either transducer#1 or transducer#2 can be excited after depositing each layer.

Among different cellular manipulation principles such as photophoresis^22,23^, magnetophoresis^24,25^ and electrophoresis^26,27^ that have been utilized to create aligned cellular arrays, acoustophoresis has emerged as a promising solution given its non-contact^28^ and label-free^29^ bulk cellular manipulation principle. In recent years, standing surface acoustic wave (SSAW)-based acoustophoresis has been extensively explored from single cell manipulation^30^ to creation of cellular patterns with highly defined spatial organizations^29,31,32^. However, in the context of 3D bioprinting, inducing a standing acoustic wave in the substrate to create the desired streaming or acoustic radiation forces for cell manipulation limits the scope of SSAW approaches for scalability across multiple layers. In this paper, we focus on the bioprinting of constructs with controllable cellular arrays utilizing standing bulk acoustic wave (SBAW) for cell manipulation. The SBAW results from the interference pattern of a laterally propagated pressure wave within a custom-designed ultrasound alignment chamber (UAC) (Fig. 1a,b). Due to the lateral propagation, the SBAW amplitude does not change across the thickness of the bioprinted constructs (along z-axis), thereby rendering this technique more appropriate for creation of multi-layered constructs with aligned cells. The open-top design of the UAC allows for synergistic integration with bioprinting wherein the bioink can be deposited within the chamber and cellular arrays ultrasonically induced before the layer is crosslinked and subsequent layer is printed. In this paper, we integrate the UAC with a commercial bioprinter (BioAssemblyBot^TM^) to study the ultrasonically induced alignment of MG63 cells in single and multi-layered extrusion-bioprinted alginate constructs. The MG63 cells were used given their prior utilization in literature as a model cell line in anisotropic orthopaedic tissue engineering research^33,34^, and alginate was used as the model hydrogel given its broad spectrum usage in biomedical research and applications^35–37^.

We start by describing an analytical model of the physics of the cellular alignment process and identifying the underlying parameters that govern the forces acting on the cells to align them at the nearest pressure nodes. The cross-patterning UAC consists of two pairs of orthogonally arranged plate-type piezo-transducers and opposing flat glass reflectors (Fig. 1a), which allows the creation of constructs with 0°–90° cellular alignment in alternating layers. After bioprinting each layer of the bioink within the chamber pre-filled with phosphate buffered saline (PBS), cellular alignment and entrapment is achieved through synergistic acoustophoresis of the cells and chemical crosslinking of the bioink (Fig. 1c–h). The acoustophoresis occurs via acoustic radiation forces from the SBAW generated by the transducers vibrating along their thicknesses at ultrasonic frequencies. The transducer excitation creates planar longitudinal acoustic wave field along the x- or y-axes in the PBS (Fig. 1d,g). The 1-D pressure distribution p(x, t) in this acoustic wave field in the bioink follows a combination of continuity and Euler’s equations as^38^

1 $$\frac{1}{{c}_{a}^{2}}\frac{{\partial }^{2}p}{\partial {t}^{2}}-(\frac{{\partial }^{2}p}{\partial {x}^{2}})=0$$

where *c*_*a*_ = longitudinal wave speed (speed of sound) in the bioink matrix (alginate). The solution to Eq. (1) can be described as a combinatorial complex harmonic expression with pressure fields generated from the transducer and reflected from the opposing glass, as

2 $$p(x,t)={P}_{transducer}+{P}_{reflector}$$

3 $${P}_{transducer}=A{e}^{j(2\pi ft-kx)}$$

4 $${P}_{reflector}=B{e}^{j(2\pi ft+kx)}$$

where *P*_*transducer*_ = pressure field along positive x-axis, *P*_*reflector*_ = pressure field along negative x-axis, A and B = complex constants, *f* = frequency of excitation of transducers (ultrasound frequency), and *k* = wave number. At the interface of the fluid contact with the transducer (*x* = 0), the pressure is given as

5 $$p(0,t)={P}_{0}{e}^{j(2\pi ft)}$$

where *P*_0_ = pressure amplitude. A normal incidence of the planar wave results in the boundary condition of zero fluid velocity at the reflector as

6 $$v(L,t)=-\,\frac{1}{{\rho }_{a}}{\int }_{0}^{t}\frac{\partial p}{\partial x}dt=0$$

where *v* = fluid velocity along x-axis, ${\rho }_{a}$ = density of alginate, and *L* = distance between the transducer and reflector surfaces. Solving Eqs (2–6) leads us to the equation for the pressure distribution in a SBAW and a constraint on the transducer-reflector distance (*L*) as

7 $$p(x,t)={P}_{0}cos(2\pi ft)cos(kx)$$

8 $$L=\frac{n\lambda }{2}$$

where *n* = number of nodes present within the SBAW in the chamber (integer value) and *λ* = wavelength of the ultrasound. The nodes of this SBAW are fixed planes parallel to the transducer and reflector surfaces, with adjacent nodal planes separated by *λ/*2. Between any two adjacent nodal planes, the SBAW exerts a radiation force^39^ (*F*_*radiation*_) on the cells within the bioink to align them at the nearest nodal plane (Fig. 1b), thereby creating planar cellular strands.

9 $${F}_{radiation}={F}_{0}sin(2kx)$$

where *F*_0_ = radiation force amplitude described as a function of the acoustic potential amplitude (*U*_0_) and the acoustophoretic coefficient (Φ) as

10 $${F}_{0}=2k{U}_{0}\Phi $$

where

11 $${U}_{0}=\frac{{{P}_{0}}^{2}V}{8{\rho }_{a}{{c}_{a}}^{2}}$$

where

12 $$\Phi =\frac{5\gamma -2}{2\gamma +1}-\frac{1}{\gamma {\beta }^{2}}$$

where *V* = cell volume, *γ* = ratio of the density of a cell to that of alginate = ${\rho }_{c}/{\rho }_{a}$, and *β* = ratio of speed of sound in a cell to that in alginate = *c*_*c*_/*c*_*a*_. For a cell near the antinodal region traversing towards the node, the equation of motion would be given as a non-linear ordinary differential equation combining the radiation force and the drag force (*F*_*drag*_) as

13 $$m\ddot{x}+{F}_{radiation}+{F}_{drag}=0$$

where

14 $${F}_{drag}=6\pi \eta r\dot{x}$$

where $\ddot{x}$ = instantaneous acceleration of the cell, *r* = radius of the cell, *η* = dynamic viscosity of the bioink, and $\dot{x}$ is the instantaneous velocity of the cells. An analytical solution for an overdamped system ($\ddot{x}$ = 0) to Eq. (13) for the instantaneous position (*x*(*t*)) of a cell traversing from a non-nodal position *x*_0_ to the node is^39^

15 $$x(t)=\frac{1}{k}{\tan }^{-1}(\tan (k{x}_{0}){e}^{\frac{-2{F}_{0}t}{3\lambda \eta r}})$$

From this, the time to migrate the cell from an initial position *x*_0_ to a final position *x*_*f*_ could be derived as

16 $${t}_{align}=\frac{3\lambda \eta r}{2{F}_{0}}\,\mathrm{ln}(\frac{\tan (k{x}_{0})}{\tan (k{x}_{f})})$$

Assuming that the cells are spherical ($V=4\pi {r}^{3}/3$) and substituting Eqs (10) and (11) into Eq. (16), the expanded form of *t*_*align*_ becomes

17 $${t}_{align}=\frac{9\lambda \eta {\rho }_{a}{{c}_{a}}^{2}}{2\pi k{P}_{0}^{2}{r}^{2}\Phi }\,\mathrm{ln}(\frac{\tan (k{x}_{0})}{\tan (k{x}_{f})})$$

Equations (16) and (17) highlight the key parameters that govern the time it takes to achieve well-defined cellular alignment. The *t*_*align*_ is inversely proportional to square of the cell radius, implying that faster cellular alignment (lower *t*_*align*_) would be observed for larger cells. Increasing *F*_0_, reducing bioink viscosity, and increasing the frequency would also reduce *t*_*align*_. However, increasing the *F*_0_ would lead to an increased velocity of the cells, thereby increasing *F*_*drag*_, which in turn would slow down the movement of the cells. An increase in *F*_*drag*_ would be more profound for highly viscous bioinks (high *η*).

**Figure 2:**
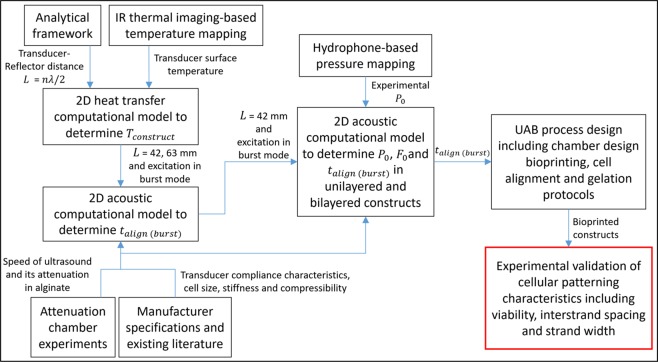
Roadmap of the constitutive studies to investigate the process physics of ultrasound-assisted bioprinting and the characteristics of bioprinted constructs.

Based on the analytical model described above, we investigate key parameters associated with ultrasound-assisted bioprinting (UAB) – excitation mode, frequency, voltage amplitude, transducer-reflector distance, and alignment time – that govern the critical quality attributes of interest within the bioprinted constructs with cellular alignment – cell viability, width of cellular strands, and spacing between adjacent cellular strands (inter-strand spacing). We also demonstrate the UAB of bilayered constructs (Fig. 1c–h) with parallel (0°–0°) or orthogonal (0°–90°) cellular alignment across layers and discuss the factors that govern the cellular distribution within such constructs. Figure 2 lays out a road map of the constitutive computational and experimental studies in this paper. These studies are performed utilizing the cross-patterning UAC, which is designed based on the rise of temperature within the construct due to the impedance heating of the transducers, and alignment time under different transducer-reflector distances and frequency – voltage amplitude combinations. To derive the temperature at the center of the construct (*T*_*construct*_) and *t*_*align*_, we develop 2D computational models in COMSOL and subsequently determine the value of *L* that satisfies Eq. (8) and keeps the temperature at or below 37 °C while achieving cellular alignment within a few minutes of the bioink being deposited. Next, we develop computational models using the relevant UAC design to estimate *F*_0_ and *t*_*align*_ that further inform the UAB process design. Finally, we experimentally validate the analytical and computational models by evaluating characteristics of constructs fabricated via UAB.

## Results

### Computational models describing the effects of UAB parameters (excitation mode, frequency, voltage amplitude, and transducer-reflector distance) on the resulting temperature rise and alignment time within bioprinted constructs

**Figure 3:**
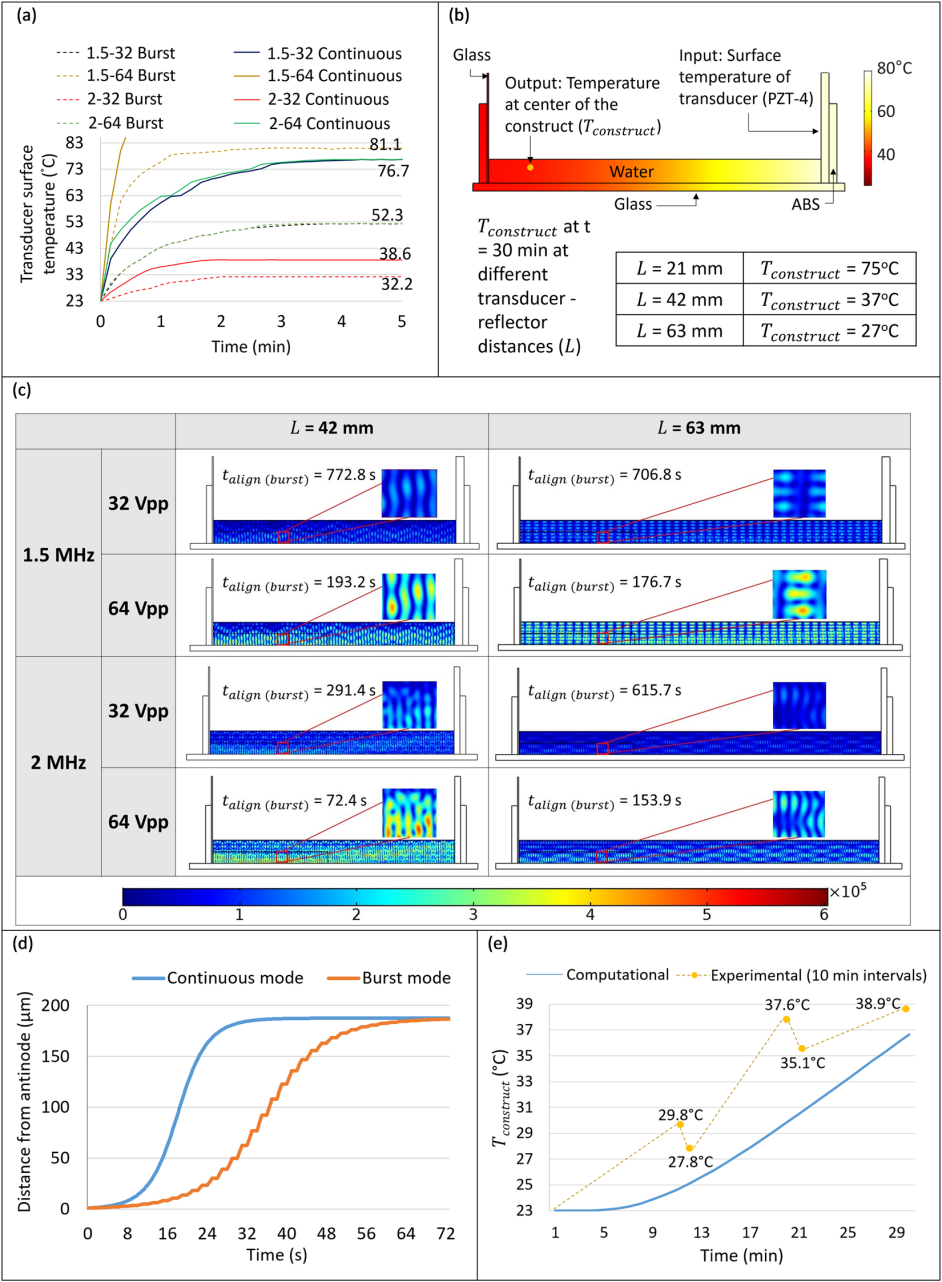
(**a**) Experimental transducer surface temperatures for the different frequency – voltage amplitude groups in burst or continuous excitation modes over time. Although the transducer was actuated for 30 min, readings for more than 5 min are not shown here since the temperature remained constant afterwards. For the 1.5 MHz–64 Vpp group in continuous mode, excitation was switched off within a few seconds because the temperature exceeded the manufacturer specification to prevent transducer compliance losses. **(b)** Materials and boundary conditions in COMSOL model to determine the temperature rise at the center region of the construct. Parametric sweep for the transducer-reflector distance (*L*) for the 1.5 MHz–64 Vpp group, with the experimental transducer surface temperatures as input, showed that the computationally estimated temperature at the center of the construct (*T*_*construct*_) remained within 37 °C at *L* = 42 mm. **(c)** Acoustic pressure distribution and corresponding cellular alignment time (*t*_*align*(*burst*)_) at *L* = 42 and 63 mm across each frequency – voltage amplitude combination in burst mode excitation. Although the peak pressure amplitude decreases by increasing *L*, cellular alignment can be achieved within 1 min of transducer excitation at *L* = 42 mm. **(d)** Computationally-estimated cellular position over time in burst and continuous excitation modes for the 2 MHz–64 Vpp group. These plots are for a cell beginning at a near anti-nodal position and migrating to a near nodal position. In burst mode, the stepwise trend is observed due to periodic excitation of the transducer. (**e**) Computationally-estimated and experimentally-measured temperatures for the 1.5 MHz–64 Vpp group in burst mode of excitation at *L* = 42 mm. The drops in temperature in experimental readings at 11 min and 20 min are due to the introduction of CaCl_2_ crosslinker solution at 23 °C. The difference between the computational estimates and experimental readings could be attributed to the homogenization of the temperature within the chamber due to fluid streaming-induced perturbations.

The design of the cross-patterning UAC to fabricate unilayered and bilayered (0°–0° and 0°–90°) constructs was based on the design constraint (Eq. (8)) and two computational studies in COMSOL to estimate – (1) temperature at the center of bioprinted construct (*T*_*construct*_) over 30 min of transducer excitation (Fig. 3a,b), and (2) cellular alignment time (*t*_*align*_) based on pressure distribution (Fig. 3c). These estimates of *T*_*construct*_ and *t*_*align*_ were determined at two excitation modes – burst (1 s burst – 1 s pause) and continuous, two frequencies – 1.5 and 2 MHz, two voltage amplitudes – 32 and 64 Vpp, and three transducer-reflector distances (*L*) that satisfied Eq. (8) for both frequencies – 21, 42 and 63 mm. Corresponding design constraints required *T*_*construct*_ to not exceed 37 °C while achieving *t*_*align*_ values that are acceptable as per the bioprinting and crosslinking protocols.

Prior to developing the computational model for determining temperature of transducer and construct, transducer surface temperature variations due to their impedance heating were experimentally assessed over time (up to 30 min) in the two excitation modes for both frequencies and voltage amplitudes. Herein, results clearly indicated that the burst mode was better at limiting the rise in temperature of the transducer surface (Fig. 3a). Especially, the temperature of the transducer in the continuous excitation mode at 1.5 MHz–64 Vpp exceeded the manufacturer-defined specification of a maximum temperature of 85 °C within 30 s, which was undesirable as it would have led to a permanent loss of compliance (deformation to applied voltage) of the transducer. In contrast, for the same frequency – voltage amplitude group in burst mode, the transducer surface temperature plateaued at an acceptable 81 °C over 30 min. Since the 1.5 MHz–64 Vpp group resulted in the highest transducer surface temperature among all burst mode groups, this group served as the upper bound and was computationally modeled to determine the *T*_*construct*_ under the three different transducer-reflector distances. In this model, a gradual surface temperature rise of the transducer up to 81 °C was inputted as an interpolation function constituting the input boundary condition (Fig. 3b). Results indicated *T*_*construct*_ to be at an acceptable 27 °C and 37 °C after 30 min of burst excitation at *L* = 63 and 42 mm, respectively (Fig. 3b). At *L* = 21 mm, the high *T*_*construct*_ (75 °C) would have been highly deleterious to the cells, and hence this design was not considered during further analysis.

Next, in order to compare between *L* = 42 and 63 mm for design optimality, we estimated the resulting alignment time in burst mode (*t*_*align*(*burst*)_) using 2-D linear acoustic models of pressure distribution along the UAC side views. To improve the fidelity of the basic model, the transducer compliance characteristics derived from the manufacturer’s specifications and the speed of sound and its attenuation in alginate solution and crosslinked hydrogel were accounted for in the model (details in the Materials and Methods section). The corresponding outputs of the acoustic computation models are presented in Fig. 3c. Note that to determine values of *t*_*align*(*burst*)_, Eq. (16) could not be used as is because it accounts for uninterrupted migration of cells (continuous mode), while in the burst mode, the cells stop migrating ($\dot{x}$ = 0) at the end of each 1 s burst. Therefore, determination of *t*_*align*(*burst*)_ required tracing the cellular position from Eq. (15) over time, with the final position of the cell at the end of each 1 s burst set as the initial position *x*_0_ for subsequent position estimations. For comparing the trends, a trajectory for cellular position in continuous mode was plotted using Eq. (15) without any modification to the initial position *x*_0_ over time. For example, the corresponding cell position plots for the 2 MHz–64 Vpp group are presented in Fig. 3d. For both these trajectories, the cell was assumed to start from a position which is 1 μm away from the antinode (*x*_0_ = (*λ*/4 − 1) µm), migrating towards its final resting position which is 1 μm away from the node (*x*_*f*_ = 1 µm). From these experimental trajectories, it was evident that the estimated *t*_*align*(*burst*)_ was twice that in continuous mode (estimated directly from Eq. (16)), as

18 $${t}_{align(burst)}=2{t}_{align(continuous)}$$

For example, at 2 MHz–64 Vpp (Fig. 3c), a cell can align in 72.4 s in the burst mode compared to 36.2 s in the continuous mode. Note that Eq. (18) is only valid for the stipulated values of *x*_0_ and *x*_*f*_, and an estimate of the cellular position at any time in burst mode may need to be derived from the plot of position versus time.

Results show that *t*_*align*(*burst*)_ is comparatively higher at 1.5 MHz than at 2 MHz, and doubling the voltage amplitude quadruples the alignment times. Regarding the relationship between *t*_*align*(*burst*)_ and *L*, according to the theory, an increase in *L* should be accompanied by a greater attenuation of the SBAW within the fluid^38^, resulting in higher estimates of *t*_*align*(*burst*)_. Computationally, this relationship was observed in the 2 MHz groups but not in the1.5 MHz groups, wherein the resulting *t*_*align*(*burst*)_ at *L* = 63 mm (706.8 s and 176.7 s at 32 Vpp and 64 Vpp, respectively) was slightly lower than that at *L* = 42 mm (772.8 s and 193.2 s at 32 Vpp and 64 Vpp, respectively). The changes in *t*_*align*(*burst*)_ due to increasing *L* are significantly higher at 2 MHz than at 1.5 MHz. In general, the *t*_*align*(*burst*)_ across all groups at *L* = 42 mm were suitable for the gradual chemical (CaCl_2_) crosslinking protocol utilized in this work. Hence, for the purposes of this study, UAC with *L* = 42 mm was further studied for computational analyses of pressure distribution and experimental analyses of cellular viability and alignment characteristics.

Finally, at *L* = 42 mm, we experimentally validated *T*_*construct*_ for the 1.5 MHz–64 Vpp group. A layer of alginate solution was bioprinted (t = 0 min), followed by the serial crosslinking protocol of adding 3 ml of 0.5%, 1% and 2% w/v CaCl_2_ at t = 1, 10 and 20 min, respectively, and the *T*_*construct*_ was measured using an infrared camera. Temperature readings (Fig. 3e) indicated that the experimentally measured *T*_*construct*_ after 30 min (38.9 °C) was close to the computational model estimate (37 °C). The slightly higher experimental *T*_*construct*_ could be attributed to fluid streaming-induced perturbations observed for the 1.5 MHz transducers^40^, which may have caused a mixing of the high temperature fluid near the transducer with the low temperature fluid in the construct region.

### Computational model describing the effects of UAB frequency and voltage amplitude on pressure distribution across multiple layers of the bioprinted construct

To derive a computational estimate of the SBAW pressure distribution and the corresponding radiation force amplitude and alignment time at each frequency – voltage amplitude combination for UAC with *L* = 42 mm in burst excitation mode, 2-D linear acoustic computational models (transducer attributes and boundary conditions described in the Materials and Methods section) depicting the pressure distribution within the top and side views of the cross-patterning UAC were developed. The top view is useful to discern the general pressure distribution and expected alignment pattern as seen from the top, while the side-view provides a representation of the pressure distribution along the construct thickness (i.e., across bioprinted layers).

**Figure 4:**
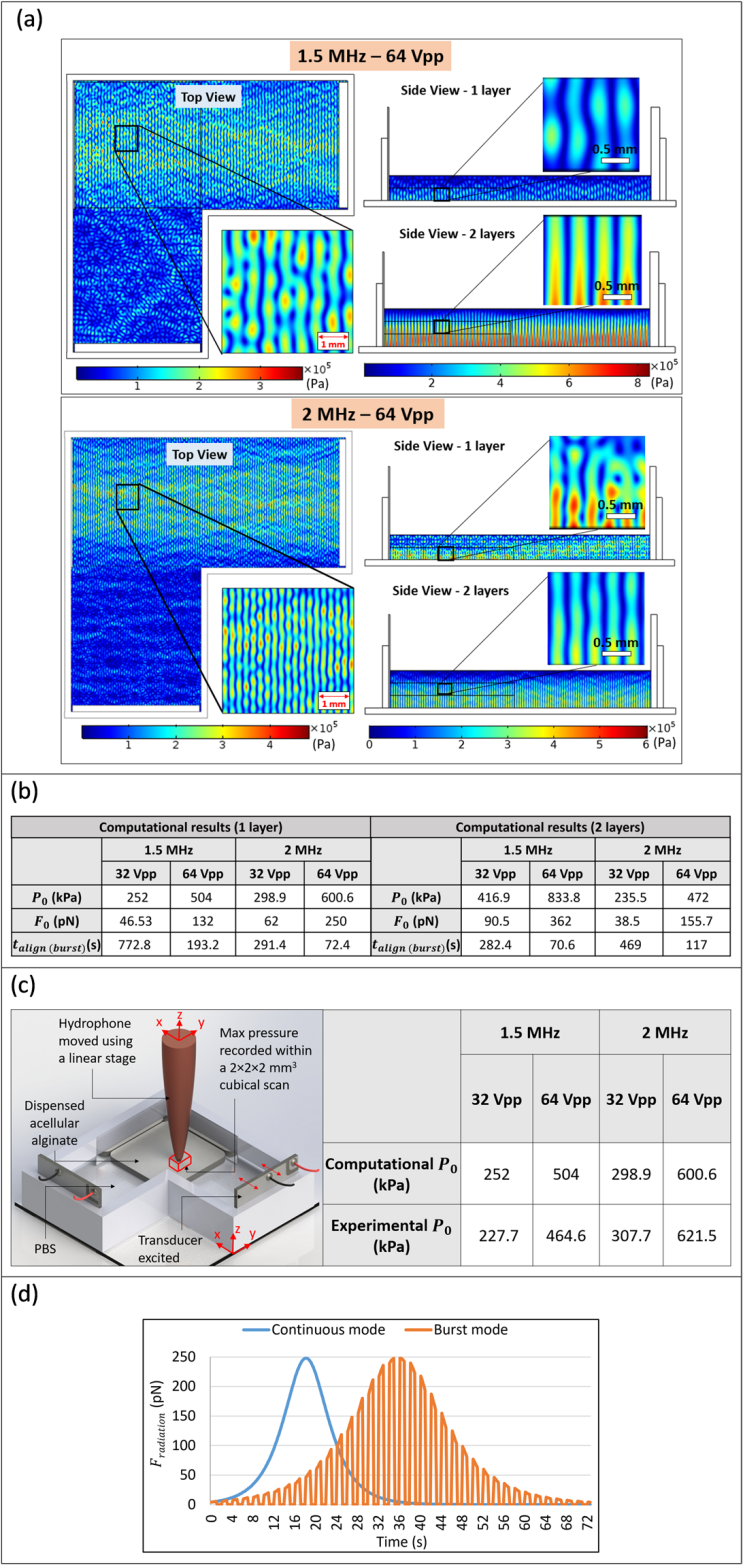
(**a**) COMSOL model outputs of the acoustic pressure distribution within the UAC for the 1.5 MHz–64 Vpp and 2 MHz–64 Vpp groups portray nodal planes parallel to the excited transducer in both top and side views. The model also accounts for the change in transducer thickness based on its resonant frequency (1.5 or 2 MHz). **(b)** The table shows the computationally-estimated peak acoustic pressure amplitude, peak radiation force and the alignment time at each frequency – voltage amplitude combination. All these values are derived from the computational models depicting pressure distribution along the side views since they account for the presence of multiple layers and a greater transducer height relative to that of the contacting fluid. **(c)** Experimental measurement of peak acoustic pressure amplitudes and their comparison with computational estimates. The experimentally-measured values are close to the corresponding computational estimates. (**d**) Computationally-estimated *F*_*radiation*_ over time in burst and continuous excitation modes for the representative 2 MHz–64 Vpp group, for a cell beginning at a near anti-nodal position and migrating to a near nodal position.

Figure 4a depicts the computational pressure distributions for the 1.5 MHz–64 Vpp and 2 MHz–64 Vpp groups. The 32 Vpp groups demonstrated identical pressure distribution patterns, but the peak pressures were half compared to corresponding 64 Vpp groups. In these outputs, the top view of the UAC (Fig. 4a) shows that the pressure nodal lines within the construct are parallel to the transducer surface, which is analogous to the theory of planar waves emanating from the transducers, resulting in a SBAW with planar nodes. For all groups, side views of the unilayered construct depict a greater pressure amplitude of the standing wave near the bottom. The same effect is also seen in both the first and the second layer of the bilayered construct. The corresponding computational estimates of peak acoustic pressure and radiation force amplitude and *t*_*align*(*burst*)_ are presented in Fig. 4b. While the pressure amplitude was directly proportional to the voltage amplitude, its relationship with frequency depended on the thickness of the construct. For the unilayered construct, the acoustic pressure was highest in the 2 MHz–64 Vpp group (direct relationship with frequency), while for bilayered constructs, it was the highest in the 1.5 MHz–64 Vpp group (inverse relationship with frequency). To experimentally validate the computational model, volumetric scans were performed across the construct using a pressure hydrophone (Fig. 4c). The experimental peak pressure amplitudes were closely correlated with corresponding computational estimates, thereby confirming the accuracy of the model in describing the physics of the process that governs the cellular alignment over time.

In addition to the previously presented plot of cellular position (Fig. 3e), the plot of *F*_*radiation*_ over time further elucidates the mechanics of cellular motion (representative plot depicted in Fig. 4d). Herein, it is assumed that *F*_*drag*_ is equal to *F*_*radiation*_ and that the cell moves at a constant speed ($\ddot{x}$ = 0). The *F*_*radiation*_ on the cell slowly increases, maximizing at a distance of *λ/4* from the antinode (or the node) and subsequently reducing as the cell approaches the node. Based on the analytical framework and corresponding computational outputs, faster alignment can be achieved by increasing the *F*_*radiation*_, even with burst mode of excitation. Since the *F*_*radiation*_ is proportional to square of the pressure amplitude, a slight increase in the voltage amplitude would significantly increase the *F*_*radiation*_ and reduce the *t*_*align*(*burst*)_. For example, doubling the voltage amplitude would quadruple the *F*_*radiation*_, thereby reducing the *t*_*align*(*burst*)_ by a factor of 4 (Fig. 4b) Moreover, introducing gradual chemical crosslinking would allow more time for the cells to migrate before being entrapped, thereby improving the anisotropy within the bioprinted constructs.

### Analysis of alignment in unilayered and bilayered constructs

Figure 1c–h illustrates the UAB process that utilizes multiple transducer-reflector pairs within the cross-patterning UAC to enable the bioprinting of constructs with aligned cells. The unilayered construct is retrieved from the chamber after layer#1 has been bioprinted and crosslinked. For the bilayered constructs, the second layer is added onto the first layer, and transducer#2 excited to achieve orthogonal (0°–90°) cellular alignment, or transducer#1 is excited (same as layer#1) to achieve parallel cellular alignment (0°–0°) in alternating layers of crosslinked constructs. Based on the computational inferences about temperature rise in the constructs and the alignment time, burst mode of excitation was used in the experimental studies in order to maximize cell viability. Following the aforementioned serial chemical (CaCl_2_) crosslinking protocol ensured that the crosslinking progressed gradually across each layer and rendered a greater degree of distinction in the cellular strands within the constructs.

**Figure 5:**
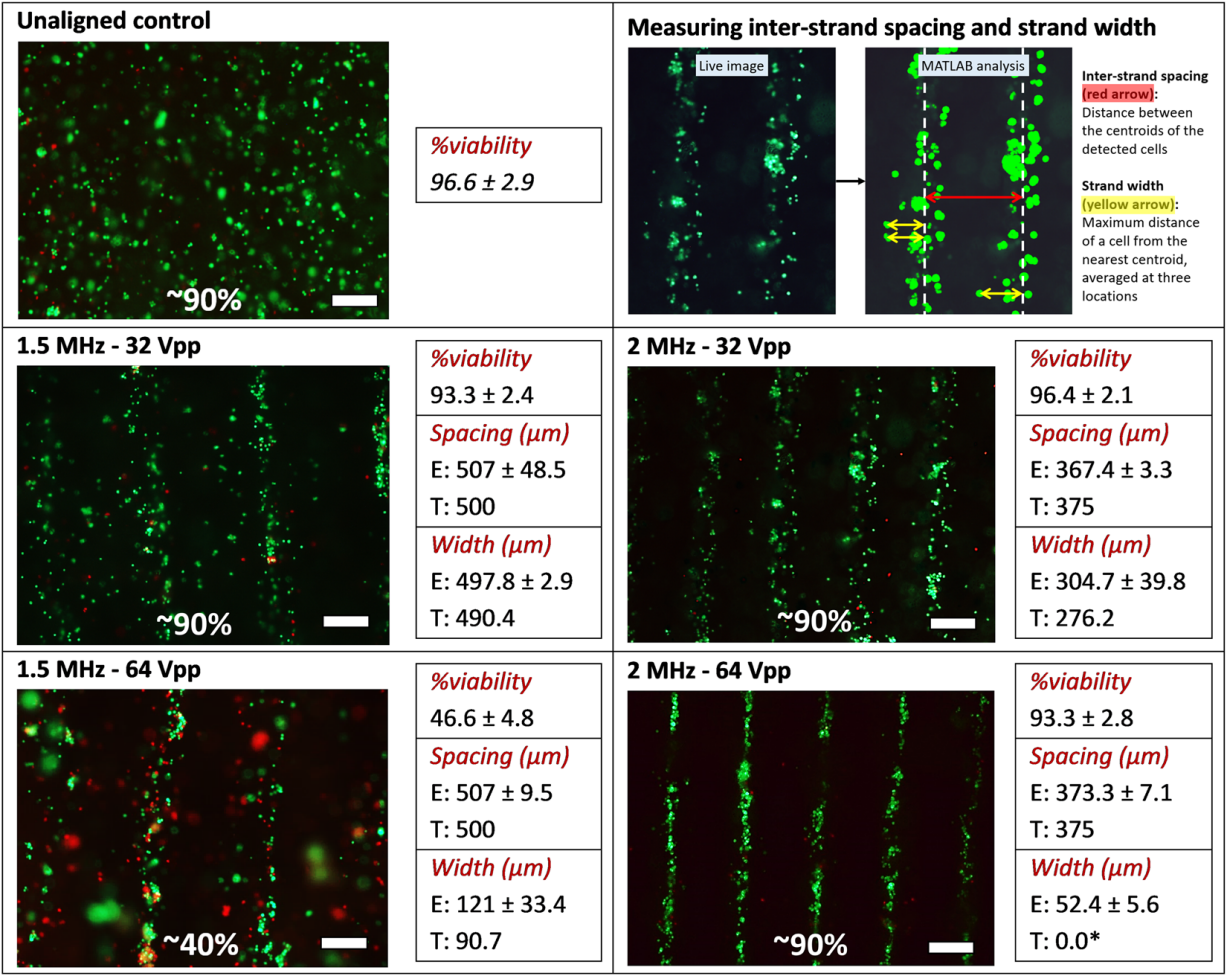
Live/Dead analyses of unilayered constructs fabricated via UAB (burst mode transducer excitation). The critical quality attributes are reported as mean ± SD. Letters E and T represent the experimental values and the theoretical estimates, respectively. Results indicate that the MG63 cellular viability is significantly affected by the interaction of frequency and voltage amplitude (p < 0.001). The viability of the 1.5 MHz–64 Vpp group was significantly lower than the other three groups (p < 0.05). The inter-strand spacing is significantly affected by only the frequency (p < 0.001) while the strand width is significantly affected by the interaction of frequency and voltage amplitude (p < 0.005). The experimental inter-strand spacing closely correlates with the theoretical estimate for each group. The experimental strand width closely approximates the range (Mean ± 1 SD) of experimental widths for all groups except 2 MHz–64 Vpp. This could be attributed to the compactness of the cellular clusters and the *F*_*secondary radiation*_ due to SBAW scattering that impedes cellular migration to the nodes. *Note that this is the estimate when only one cell is present in the bioink, and it could change when other cells are already present at the nodes. Scale bar = 250 µm.

Figure 5 illustrates the Live/Dead images and the corresponding cell viability, inter-strand spacing and strand width from the MATLAB image analysis of the unilayered constructs for the four frequency – voltage amplitude combination groups. The interaction of frequency and voltage amplitude significantly affected the MG63 cell viability (p < 0.001). The 1.5 MHz–64 Vpp group demonstrated a significantly lower viability compared to other groups as well as its corresponding control. This could be attributed to the higher observed *T*_*construct*_ due to viscous streaming induced perturbations as explained in the first section of the results. The inter-strand spacing was significantly affected only by the frequency (p < 0.001), and the experimental values closely correlated to the theoretical spacing of λ/2 at each frequency. As such, it is clearly evident that the inter-strand spacing can be controlled to a high degree of accuracy by controlling just the frequency.

The experimental strand width was significantly affected by the interaction of frequency and voltage amplitude (p < 0.005), with each group significantly different from others (post hoc results). To theoretically model the strand width (*w*), it was assumed that a cell beginning at the antinode would reach a distance of *w*/2 from the node until the time the crosslinking of alginate solution impedes any further cellular migration (*t*_*crosslinking*_). The *t*_*crosslinking*_ was estimated to be 2 min based on experimental observations with our gradual crosslinking protocol. Since the unilayered constructs were fabricated in burst mode, it was essential to trace the cellular position (Eq. (15)) over time (e.g., Fig. 3d) and estimate the width as twice the cellular position (distance from node) at *t*_*crosslinking*_ = 2 min. For every group except 2 MHz–64 Vpp, the theoretically-estimated width lied within the range (mean ± SD) of the measured experimental width. These results indicate that the overdamped model (Eq. (15)) can be a good approximation of the cellular position over time, provided the cellular positions are accurately traced over time. The difference between theoretically-estimated and experimentally-measured width in the 2 MHz–64 Vpp group can be attributed to the effects of cellular compaction and secondary radiation forces. At 2 MHz–64 Vpp, the *t*_*align*(*burst*)_ is less than the *t*_*crosslinking*_, which results in a theoretical width estimate (corresponding to twice the distance between center of the cell’s final resting position after *t*_*crosslinking*_ and the nearest node, assuming that the starting position of the cell was at the antinode when the transducer was excited, when only a single cell is present in the bioink) of 0 µm. However, this translates experimentally to a highly compact clustering of the cells, wherein any motion of the cells is impeded by the presence of other cells closer to the node. This results in higher experimental width estimates (double the maximum distance of the cells from their nearest node). This is not the case for other groups, where the *t*_*align*(*burst*)_ is higher than *t*_*crosslinking*_. Additionally, the presence of cells already at or near the node could be expected to cause scattering of the SBAW, that would result in an opposing secondary radiation force^41^ (*F*_*secondary radiation*_) acting on another cell traversing towards the same node, thereby impeding its transition. For the cellular migration within the alginate in this study, *F*_*secondary radiation*_ can be estimated as

19 $${F}_{secondaryradiation}=\frac{4\pi {E}_{0}{k}^{2}{r}_{1}^{3}{r}_{2}^{3}{k}_{1}{k}_{2}}{9{k}_{a}{d}^{2}}$$

where *r*_1_ = *r*_2_ = radius of the cell 1 (present at the node) and cell 2 (migrating to the node), respectively, *k*_1_ = *k*_2_ = *k*_c_ = compressibility of the cells, d distance between cell 1 and 2, and *k*_*a*_ = compressibility of alginate solution. At all frequency – voltage amplitude groups except 2 MHz–64 Vpp, *F*_*secondary radiation*_ was at least six orders of magnitude lower than *F*_*radition*_ (primary radiation forces in Eq. (9)) at t = 2 min. For the 2 MHz–64 Vpp group, however, the magnitude of *F*_*secondary radiation*_ exceeded the primary radiation force magnitude at 72 s. This effect would be further compounded due to the presence of other cells near the node. This also explains why the mean experimental width for each group is comparatively larger than the corresponding theoretical estimate. Another point to note is that as the magnitude of *F*_*secondary radiation*_ approaches that of *F*_*radition*_, Eq. (15) loses its validity for describing cellular position as a function of time. Therefore, future studies would need to account for these secondary radiation forces, especially at larger cellular concentration or longer *t*_*crosslinking*_ compared to *t*_*align*_, to achieve a high degree of fidelity in estimating the resulting cellular distribution characteristics.

**Figure 6:**
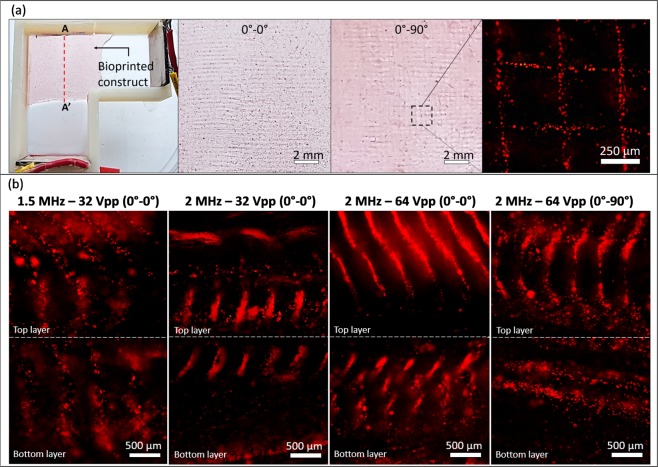
(**a**) Macroscopic and **(b)** microscopic fluorescence images of neutral red-stained MG63 cellular alignment within bilayered constructs as viewed from the top and across a side section. The images of 1.5 MHz–64 Vpp group have not been shown because the alignment was not visible due to low fluorescence (resulting from a poor cell viability) and heavy perturbations.

Figure 6 depicts the top view and side views of the bilayered constructs with parallel (0°–0°) and orthogonal (0°–90°) cellular alignment across layers. The microscopic images for the side views (Fig. 6b) depict varying degree of alignment within each group. While a high degree of 3D alignment is evident in groups with higher acoustic pressures, there is a typical tilting of the strands, which is greater for the top layer. This could be attributed to the viscous streams at the initiation of each excitation burst, wherein the PBS is scattered away from the transducer every 1 s. For each construct, the top layer exhibits a greater degree of 3D alignment, while the bottom layer exhibits a greater degree of alignment near the top. This could be attributed to a greater compliance of the transducer surface near its top region as compared to its bottom^40^. This non-uniform compliance could also explain the increased tilting of cellular arrays in the top layer. Within the top layer of the 2 MHz–32 Vpp construct, there is a greater degree of alignment at the bottom of the layer. This could be a result of the higher radiation force near the bottom of the top layer as predicted by the computational model (Fig. 4a). The image for the side view of the 1.5 MHz–64 Vpp construct is not shown here because the large amount of perturbations, large radiation forces, and exposure to high temperatures for 60 min lowered the cell viability significantly, thereby lowering the neutral red fluorescence below detectable levels. It should also be noted that some artifacts in the constructs and images resulted from slicing procedures, non-homogenous crosslinking, and fluid streaming-induced perturbation effects, which were more pronounced for layers at greater z-heights^40^.

## Discussion

Bioprinting enables the fabrication of engineered tissues with tissue- and patient- specific geometries using living cells as part of the manufacturing process^1–5^. While typical bioprinting processes can reproduce the macro-architectural characteristics of the tissue based on computer-aided reconstructions of medical images, it is essential to also mimic the intrinsic micro-architecture of the cells and ECM components to achieve the tissue-specific functional properties. To achieve this, acoustophoresis utilizing bulk acoustic waves offers a label-free, non-contact and non-deleterious bulk cellular manipulation mechanism to rapidly create 3D cellular patterns within the bioprinted constructs. Although there are other techniques that can be utilized to manipulate cells into aligned patterns, these may require addition of chemical (chemotaxis^42,43^) or magnetic (magnetophoresis^44,45^) labels to cells or the biomaterial, or complex setups which can limit scalability (electrophoresis^26,27^ or photophoresis^46,47^). They may also be constrained by the cell size that can be manipulated (usually *ϕ* < 10 µm for photophoresis^48^). In the context of acoustophoresis, standing surface acoustic waves (SSAW) have been investigated in literature for sorting^29,49^, mixing^31^, patterning^30,32^ and transportation^30^ of cells. However, SSAW-based techniques are typically limited to 2D cell manipulation, mostly for microfluidic applications^50–52^. For 3D manipulation required for scalability and application towards bioprinting, the radiation pressures in SSAW-based techniques are inadequate, especially as the thickness of the hydrogel increases due to layer addition, and the cells to be manipulated are farther from the vibrating substrate. Accordingly, this study proposes SBAW as an effective alternative for 3D cell manipulation in the context of bioprinting. The lateral propagation of the pressure wave in SBAW enables cell manipulation across the thickness of the hydrogel constructs (for example, across both layers in Fig. 4a,b).

In this work, we have synergistically integrated bioprinting and acoustophoresis to fabricate single or multi-layered anisotropic cellular constructs and investigated the effect of key UAB process parameters – excitation mode, frequency and voltage amplitude – on the cellular viability and alignment characteristics. We have used sodium alginate as a model biopolymer due to its wide usage in tissue engineering^36,53^ and bioprinting^35,54^ literature. Importantly, the viscosity of the alginate solution is greater than that of other hydrogel solutions (non-crosslinked form) commonly used for bioprinting^13,55^. To assist in further development of biofabrication strategies incorporating acoustophoresis to render alignment within different types of bioinks and applications, we discuss a framework for optimizing the UAC design and process-structure interactions for achieving high cellular viability and highly controllable cellular inter-strand distance and cellular strand width within the bioink.

To minimize any adverse effects on cell viability, an important factor to consider is the increase in temperature due to impedance heating of the transducer. Minimizing the temperature induced cell lysing could be ensured by optimizing process parameters (e.g., reducing voltage amplitude or *t*_*crosslinking*_), preventing direct contact of the transducer with the bioink, reducing transducer impedance, utilizing heat shielding or re-designing the UAC based on computational model similar to the one discussed in this study. An example of re-designing could entail increasing the transducer-reflector distance in the chamber so that the bioprinting region is farther away from the transducer. In doing so, however, one would also have to consider the reduction in pressure amplitude and its effect on alignment time, and proportionally vary the UAB process parameters and crosslinking protocols. For better modelling of the temperature rise, a computational model that accounts for temperature harmonization due to fluid streaming-induced perturbations may also need to be investigated in future. In general, these perturbations could be reduced by reducing the pressure amplitude, increasing the transducer-reflector distance, and improving the transducer compliance. In this study, the rise in the temperature at the center of the construct is reduced by the addition of CaCl_2_ crosslinker solution at room temperature at each 10 min interval. This might not be the case when a photo-crosslinkable hydrogel such as GelMA is used, wherein uninterrupted increase in temperature may lead to reduction in viability. This effect would be further exacerbated by streaming-induced perturbations and temperature shocks at higher duration or voltage amplitude of excitation^40^. However, since the viscosity of the liquid GelMA at 37 °C is lower than that of the alginate used in this study^56^, comparatively lower pressure amplitudes can be used to induce alignment. Furthermore, photo-crosslinking of GelMA is faster^57^ than the chemical crosslinking of alginate in this study. Together, these would alleviate the issue of temperature homogenization-induced reduction in viability. Furthermore, selective crosslinking methods such as stereolithography^58^ and digital light projection^59^ could be used to create high fidelity GelMA constructs. The changes in temperature due to in-process impedance heating of the transducer would also be critical for UAB of thermally crosslinkable hydrogels such as collagen, in that, they will play a role in their crosslinking. Towards this, the road map set forth in this work can be used to optimize the transducer-reflector distance, mode of excitation, voltage amplitude, and excitation duration, so that the transducer heating can be used advantageously to crosslink the collagen while allowing sufficient time for the cells to align within the construct without affecting their viability.

The cell viability may also be affected by acoustic cavitation effects which occur when dissolved gas microbubbles expand and collapse within the standing wave-field, resulting in a localized rise in temperatures and abrupt changes in pressure fields. These effects are enhanced (i.e., formation of larger bubbles) at lower frequencies, larger pressure amplitudes and for longer duration of ultrasound^60^, and may lead to rupturing of cell membranes^61^. Prolonged exposure to cavitation effects, increased cavitation density (number of bubbles per unit volume) and larger bubble sizes may cause irreversible damage to the cells and affect their long term viability. Moreover, cavitation effects may also cause turbulence in the system^60^, thereby disrupting any cell alignment. Cavitation effects can be minimized by using lower voltage (pressure) amplitudes and reducing the duration of ultrasound^60^. Shorter but frequent bursts of ultrasound may also lead to smaller bubble formation^60^, thereby minimizing the effects of acoustic cavitation. Alternatively, appropriate surfactants could be used to reduce coalescence between bubbles, thereby reducing their size^60^.

For applications requiring a high degree of control over the spatial distribution of the cells, an optimization of the excitation mode, excitation duration, frequency, voltage amplitude, and crosslinking protocol would be required. The cellular inter-strand spacing could simply be controlled by varying the ultrasound frequency. For the cellular strand width however, the duration of excitation should be determined so that a cell starting at the antinode halts at a distance *w*/2 from the node. This could be calculated using Eq. (20) for a continuous excitation mode (*w*_*continuous*_), or by plotting the cellular position over time for a burst excitation mode, after the radiation force magnitude has been derived from the computational model discussed in this work.

20 $${w}_{continuous}=\,2(x({t}_{crosslinking}))=\frac{2}{k}{\tan }^{-1}(\tan (k{x}_{0}){e}^{\frac{-2{F}_{0}{t}_{crosslinking}}{3\lambda \eta r}})$$

where *x*_0_ and *t*_*crosslinking*_ are user defined (assumed to be λ/4 − 1 µm and 2 min, respectively, for the present work). Moreover, at higher cellular concentrations and in processing scenarios where *t*_*align*_ (burst or continuous) is less than *t*_*crosslinking*_, cellular compaction and *F*_*secondary radiation*_ will need to be considered, which may increase the derived theoretical width estimates. In this study, it was experimentally observed that a high concentration of CaCl_2_ solution disturbed the cell alignment, as it led to greater contraction of the hydrogel due to faster crosslinking. Thus, in order to achieve a high structural fidelity, the aforementioned gradual crosslinking protocol was utilized. However, this protocol made the estimation of *t*_*crosslinking*_ difficult. This may not be an issue for other bioinks such as the photo-crosslinkable ones that utilize faster crosslinking modalities, which would then yield a higher degree of control over the strand width.

Within bilayered constructs, it was evident that a higher voltage amplitude resulted in higher fidelity in 3D. However, non-uniform transducer compliance^40^ may introduce greater tilting of arrays at greater z-heights. To reduce tilting of arrays, the voltage amplitude could be reduced with increasing z-height (e.g., addition of a layer), while still maintaining a 3D alignment throughout the thickness of the construct. It was also observed that the orientation of the SBAW in the second layer does not have an effect on the alignment within the first layer, since the viscosity of the crosslinked alginate is considerably higher than that of the alginate solution. Thus, to change the orientation of cellular alignment between layers, each layer can be crosslinked to entrap the cells, followed by the excitation of a transducer with the orientation of interest for the subsequent layer. Another important aspect is that, since the waves emitted from the transducer are planar, an array of transducers-reflector pairs could be devised with varying orientation such that the planar SBAW generated by one transducer-reflector pair does not interfere with that from another^40,62^. While the transducers were arranged in orthogonally and excited one at a time in this work, future studies could investigate UACs with different transducer orientations or simultaneous excitation (with or without phase lag) to achieve unique organizational patterns^40,62,63^.

In the present study, some of the confounding factors (e.g., transducer surface temperature and fluid streaming-induced perturbations) were characteristics of the commercial transducers used. Future studies can investigate alternative transducer designs or fabrication methods to minimize impedance losses and achieve more uniform surface compliance. Future studies can also investigate the UAB of aligned multi-cellular and multi-material constructs designed for tissue-specific applications, and study their maturation in culture over time. These longer term studies would require the use of highly macro-porous hydrogels, incorporation of vasculature, and perfusion bioreactors to facilitate nutrient and waste transport, thereby improving the resultant tissue characteristics. In addition to cell viability, other functional responses of cells (e.g., gene and protein expressions) as well as biomechanical characteristics of aligned constructs would also need to be investigated.

## Materials and Methods

### UAC design and fabrication

To create the cross-patterning UAC (Fig. 1a), first, the acrylonitrile butadiene styrene (ABS) casing was 3D printed (uPrint SE Plus, Stratasys, Eden Prairie, MN) and attached to a 100 cm^2^ sterile cell culture dish (Thermo Fisher Scientific, City, State). Plate-type piezo transducers, with Tegaderm^®^ (3M Technologies, St. Paul, MN) wrapped around the surface for biocompatibility, were then attached to the chamber. Opposite to each transducer, Corning^®^ cover glass (Sigma-Aldrich, St. Paul, MN) was attached, that acted as the reflector. Although the transducer thickness varied at each resonant frequency as per manufacturer’s specifications, a transducer-reflector distance (*L*) of 42 mm was used to satisfy Eq. (8) while ensuring that the final temperature within the constructs did not exceed 37 °C. Each transducer was actuated through a high frequency amplifier (Electronics & Innovation Ltd., Rochester, NY) that received the sinusoidal burst or continuous voltage signal from a function generator (Keysight Technologies Inc. Santa Rosa, CA).

### Characterizing acoustical properties of alginate solution and hydrogel

**Figure 7:**
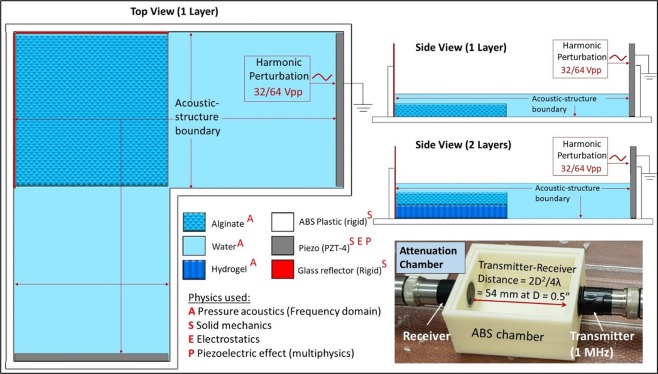
Boundary conditions and interfaces in the COMSOL model of the cross-patterning UAC for the top view and unilayered and bilayered side views. Bottom right image shows the custom-designed attenuation chamber used to measure the speed of sound and its attenuation in the alginate solution and hydrogel (after crosslinking).

All components of attenuation testing chamber (Fig. 7) were 3D printed out of ABS. Then, 1 MHz unfocused immersion transducers (emitter and receiver) (Olympus Instruments, Tokyo, Japan) were attached to either holes of the chamber. The distance between the transmitter and the receiver (*L*_*TR*_) equaled

21 $${L}_{TR}=2{D}^{2}/4\lambda $$

where *λ* = 1.5 mm in water at 1 MHz, and *D* = 0.5 in for the transducer. This ensured that a planar wave was received at the receiver end. For determining the acoustical properties of alginate solution, the chamber was filled with 120 ml of alginate solution and transducer excited with sinusoidal voltage signal at 1 MHz and 1 Vpp amplitude using a function generator (Keysight Technologies Inc. Santa Rosa, CA). To determine the acoustical properties of the crosslinked alginate, the chamber was filled with 120 ml of PBS, and a 25 mm thick crosslinked alginate construct (crosslinked using the same protocol used for the UAB constructs) was placed in the middle of the chamber. The signals propagating through the alginate as well as the PBS were acquired by using a Picoscope^®^ (5000 series, Pico Technology, St Neots, UK) with sampling frequency of 17.8 GHz. The attenuation was measured based on a substitution approach from established literature^64^, and the speed of sound was measured based on time of flight of the signal propagated in the medium (alginate or PBS) between the transmitter and receiver.

### Computational model for determining SBAW pressure distribution

To assess the SBAW pressure distribution, 2-D linear acoustic model of the top view and side view of the cross-patterning chamber were setup in the acoustic-piezoelectric interface in COMSOL Multiphysics^®^ (Comsol Inc., Burlington, MA). The material and boundary attributes have been shown in Fig. 7. The out of plane thickness for the solid mechanics and electrostatics modules was 20 mm for the side views and 3 mm for the top view. To accurately model the transducer deformation under the applied voltage signal, stress-charge form of transducer compliance was used. It follows the equation

22 $$T={c}_{E}S+{e}^{T}E$$

where *T* = stress,*E* = electric field, *c*_*E*_ = stiffness and *e*^T^ = transducer coupling constant (product of the piezoelectric constants). Material ZX plane system was used as the transducer reference frame with axis#3 along the thickness and axis#1 and 2 along the surface. All the variables in Eq. (22) were defined as vector notations in COMSOL with *c*_*E*(11)_ = *c*_*E*(22)_ = 86 GPa, *c*_*E*(33)_ = 73 GPa, *c*_*E*(66)_ = 172 GPa, ${e}_{(31)}^{T}$ = ${e}_{(32)}^{T}$ = −12.4 C/m^2^, ${e}_{(33)}^{T}$ = 23.36 C/m^2^. To the transducer, an isotropic structural loss factor of 1/1800 and a dielectric dissipation factor of 0.4 was also applied. All these attributes were derived from the manufacturer’s specifications^65^. The attenuation of ultrasound in PBS (α_PBS_ in dB/cm) was assumed to be same as that of water, given by^66^

23 $${\alpha }_{PBS}={\alpha }_{water}={\alpha }_{0}{f}^{2}$$

where *α*_0_ is 2.2 × 10^−15^ dB/cm.Hz^−2^ for water and *f* is 1.5 or 2 MHz. A similar frequency-dependence model was used to determine the attenuation from the reference value at 1 MHz within the alginate solution and hydrogel derived from the attenuation chamber experiments (Fig. 7), with *α*_0_ being 69.2 × 10^−15^ and 348 × 10^−15^ dB/cm.Hz^−2^, respectively. The speed of sound in alginate solution and its hydrogel were experimentally determined to be equal to 0.998 times the speed of sound in water (*c*_*water*_). Thus, for the computational model, *c*_*a*_ = *c*_*water*_ = 1500 m/s was utilized. The density and viscosity of the alginate solution were determined to be *ρ*_*a*_ = 1.015 g/cm^3^ (calculated from experimental weight and volume measurements) and 70 cP (from rheological experiments), respectively, and its compressibility^67^ was assumed to be ${k}_{a}$ = $1/{\rho }_{a}{c}_{a}^{2}$ = 4.37 × 10^−10^ Pa^−1^. The cells were assumed to be spherical with radius 12 µm (from microscopic measurements as well as literature^68^), and the compressibility^69^ and speed of sound in cells^70^ were assumed to be *k*_*c*_ = 4 × 10^−12^ Pa^−1^ and *c*_*c*_ = 1530 m/s, respectively. All other material properties were used from the in-built material library of COMSOL or from manufacturer’s specifications, and remaining boundary conditions were the default values in COMSOL. The entire geometry was assigned a free triangular mesh with maximum element size of 0.0375 mm (𝜆/20 at 2 MHz) to ensure a reliable output^62^. The computational times in a PC with 16 GB graphics and 32 GB RAM for top view, unilayer side view and bilayer side view were 6 min, 1 min and 1.5 min, respectively.

### Computational model for estimating temperature rise in the construct and correlation with experiments

The design of the cross-patterning chamber was influenced by the temperature rise within the chamber due to the heating of the transducer. This rise in chamber temperature was predicted through a 2D computational model setup in COMSOL using the heat transfer in fluids module. The corresponding material attributes and the boundary conditions have been detailed in Fig. 3b. Briefly, the default attributes within COMSOL stored for the materials PZT-4, water, glass and ABS were used to define the transducer, fluid, and substrate and chamber regions, respectively. As an input boundary condition, the temperature rise within the transducer derived through experimental measurements using IR thermal imaging tool (E5, FLIR Systems, Wilsonville, OR), was added as an interpolation function at the transducer surface in contact with the fluid. An open boundary was defined at the fluid-air interface. A free triangular mesh with maximum element size of 0.1 mm was defined for the model, which resulted in a computational time of 30 sec. Plots of the resultant *T*_*construct*_ over time were generated (Fig. 3e). In order to determine correlation with the results of the computational model, constructs were bioprinted and actual *T*_*construct*_ measurements conducted using the IR thermal imaging tool at 10 min intervals alongside the gradual crosslinking process (Fig. 3e).

### MG63 cell culture and bioink preparation

The alginate-matrix based bioink contained homogeneously suspended MG63 cells at a density of 10^6^ cells/ml. First, cryopreserved MG63 (ATCC, CRL^®^-1427^TM^) cells were quickly thawed at 37 °C and cultured in T-75 flasks (Nunc^TM^ Easy Flask^TM^, Thermo Fisher Scientific) (250,000 cells in 13 ml of media per flask). The media comprised of 90% v/v MEM without L-glutamine (M2279, Sigma Aldrich) and 10% v/v FBS (10438018, Thermo Fisher Scientific) and was changed every 48 hours until 80% confluency. For creating constructs with red-stained cells for fluorescence and brightfield imaging, the culture media in the 80% confluent T-75 flasks was replaced with media containing 0.001% w/v neutral red dye (Sigma Aldrich) followed by incubation of the flasks for 1 h. The dyed or non-dyed cells were passaged using TrypLE select 1X (Thermo Fisher Scientific) and centrifuged at 120 g for 5 min to create a cell pellet. The bioink matrix was 2% w/v sodium alginate (Manugel® GMB, DuPont, Wilmington, DE) solution in PBS (Sigma Aldrich), autoclaved at 16 psi and 121 °C for 30 min (BioClave 16, Benchmark Scientific Inc., Sayreville, NJ) (resultant molecular weight M_n_ = 67 kDa). The cell pellet was homogeneously suspended in the alginate solution at 1 × 10^6^ cells/ml to formulate the bioink.

### Creation of unilayered and bilayered constructs (0°–0° and 0°–90°) with aligned cells

To create each construct, the cross-patterning chamber was first disinfected by swabbing with 70% ethanol and UV exposure in the biosafety cabinet for 30 min. The chamber was then setup within a BioAssemblyBot^TM^ (Advanced Solutions Life Sciences, Louisville, KY). For the unilayered or bilayered constructs, bioink with unstained or neutral red-stained cells was utilized, respectively. During bioprinting, 800 µl of bioink (resulting in a 2 mm thick layer with 20 × 20 mm^2^ section) was printed in the form of adjacent strands through the syringe extruder via a 25 G nozzle at 2.2 psi extrusion pressure and print speed of 10 mm/s in the UAC prefilled with 5 ml of PBS. For the unilayered construct, printing was stopped after first layer, while bilayered construct constituted a 30 min pause in between the layers. After stopping or pausing, transducer#1 (Fig. 1c) was vibrated along its thickness (t = 0 min) using a sinusoidal voltage signal in burst mode, to create the SBAW and render cellular alignment. As alignment progressed, bioink was gradually crosslinked by addition of 3 ml of 0.5%, 1% and 2% w/v CaCl_2_ at t = 1, 10 and 20 min, respectively, to minimally disturb the cellular alignment. The presence of PBS was necessary to achieve homogeneous distribution of the gelation ions across the alginate constructs, and our material formulation and protocols ensured that there was no diffusion of the alginate into PBS. For the second layer in bilayered constructs, the supernatant PBS solution with CaCl_2_ was aspirated and 5 ml of fresh PBS added. Second layer of bioink was then printed over the first layer using the same bioprinting parameters and voltage signal applied to transducer#1 (0°–0° construct) or transducer#2 (0°–90° construct). The gradual crosslinking protocol was same as the first layer. For all constructs, the voltage signal was switched off after 30 min of crosslinking time per layer, and the crosslinked construct was transferred to a six well plate and analyzed for cellular viability and alignment characteristics.

### Qualitative analysis of unilayered and bilayered cellular constructs

At each combination of frequency (1.5 and 2 MHz) and voltage amplitude (32 and 64 Vpp), unilayered constructs (n = 3) were fabricated for quantitative analysis of cellular viability, inter-strand spacing, and cellular strand width. An equal number of corresponding control samples (cellular constructs not subject to SBAW) were created in tandem. A single bilayered sample was fabricated for its qualitative analysis. Post-UAB, all constructs were incubated at 37 °C and 5% CO_2_ for 3 h before further analysis, to allow cell recovery from previous processing steps.

After 3 h, the cell viability was assessed by LIVE/DEAD^®^ assay (Life Technologies, Carlsbad, CA). For each treatment or control construct, 1 ml of PBS containing 0.5 μl calcein AM and 2 μl EthD-I was added, followed by incubation for 30 min. Subsequently, each construct was imaged at three random locations using a fluorescence microscope (DM5500B, Leica Microsystems, Wetzlar, Germany), thereby resulting in N = 9 images per treatment or control group.

A custom contrast-based MATLAB code was utilized to measure average inter-strand spacing and the average strand width^40^. The output of the code and the corresponding response measurement has been described in Fig. 5. The code detected pixels corresponding to cells within a user-defined bounding box that encompassed two adjacent strands within each image. The x-axis distance between the centroids of the detected pixels within the two strand constituted the inter-strand spacing, while twice the maximum x-axis distance between the detected pixels of the cells and the nearest centroid constituted the width measurements. This width was averaged out over three random locations within each image. Three images per construct and three constructs per frequency – voltage amplitude combination resulted in a total of 3^3^ = 27 data points per treatment group.

### Hydrophone scans to determine pressure amplitude in the cross-patterning chamber

The maximum pressure amplitude was determined from a volumetric pressure scan of the cross-patterning chamber using a hydrophone (HGL0200, Onda, City, State). First, a single layer of 800 µl alginate solution was deposited within the cross patterning chamber pre-filled with 5 ml PBS using the previously described bioprinting protocol, and the hydrophone tip (sensor) was immersed within the alginate. Then, the transducer was excited in short bursts and the hydrophone traversed across a defined volume (a cube of side 2 mm) in 0.1 mm steps along each axis to obtain a 3D volumetric scan (Fig. 4d). Herein, the derived maximum voltage at the output of the transducer was processed in a custom MATLAB program to derive the maximum pressure amplitude at that frequency – voltage amplitude combination. Due to the orthogonality of the tip of the hydrophone with respect to the direction of propagation of the acoustic wave, the voltage at the output of the hydrophone was attenuated by a factor of 3.125 (as per manufacturer specifications for our hydrophone model, which indicates that the measured voltage is 32% of the actual voltage for orthogonal orientation). Accordingly, a corresponding amplification factor of 3.125 was applied on the output voltage of the hydrophone to get the actual voltage reading (and thus the actual pressure value).

### Statistical analysis

For the unilayered constructs (n = 3), a full factorial two-way ANOVA with Tukey post-hoc tests was used to assess the effect of the treatment factors (voltage amplitude and frequency) on each outcome metric of interest. For viability analysis, student’s t-tests were also conducted to compare the treatments to the corresponding controls. All tests were performed in JMP^®^ (SAS, Cary, NC) at a significance level of α = 0.05.
